# Preclinical and experimental evidence of salvianolic acid B in the treatment of neurological diseases

**DOI:** 10.3389/fphar.2025.1606146

**Published:** 2025-06-27

**Authors:** Shijun Bi, Shibing Liu, Kunyuan Zhu, Dandan Gao, Ligang Chen, Chunyong Yu, Guobiao Liang

**Affiliations:** ^1^ Department of Neurosurgery, General Hospital of Northern Theater Command, Shenyang, China; ^2^ Department of Neurosurgery, The Eleventh People’s Hospital of Shenyang, Shenyang, China

**Keywords:** salvianolic acid B, neurological diseases, anti-inflammatory, anti-oxidative stress, neuroprotective, angiogenesis, signaling pathways

## Abstract

**Background:**

Neurological diseases such as stroke and Alzheimer’s disease pose increasing challenges to global public health. Salvianolic Acid B (SalB), a major active component of *Salvia miltiorrhiza*, has garnered attention due to its anti-inflammatory, antioxidant, neuroprotective, and pro-angiogenic properties in neurological disease treatment.

**Purpose:**

This paper aims to review the mechanisms and effects of SalB in the treatment of neurological diseases, exploring its role in improving neurological function, mitigating neuroinflammation, and reducing oxidative stress.

**Results:**

SalB demonstrates multifaceted mechanisms in neurological disease management. In animal models of cerebral ischemia/reperfusion injury, SalB reduces infarct size and enhances neurological recovery via anti-inflammatory, anti-oxidative stress, and angiogenic pathways. It protects the blood-brain barrier and inhibits neuronal apoptosis in stroke models. In spinal cord injury models, SalB alleviates edema and promotes motor function recovery. In Alzheimer’s disease models, SalB suppresses amyloid-beta formation and neuroinflammation. Additionally, SalB exhibits antidepressant and analgesic effects in pain-depression comorbidity models. These effects are mediated through the regulation of signaling pathways, including NF-κB, AMPK, PI3K/Akt, and Nrf2, highlighting SalB’s broad therapeutic potential in neurological diseases.

**Conclusion:**

SalB exhibits promising prospects in the treatment of neurological diseases. However, its clinical application faces challenges such as chemical stability and bioavailability. Further research on the mechanisms of SalB and innovative drug delivery strategies is needed to advance its application in neurological disease therapy.

## 1 Introduction

Neurological diseases, including ischemic stroke, Alzheimer’s disease (AD), Parkinson’s disease (PD), multiple sclerosis, spinal cord injury and vascular dementia, are among the most prevalent and debilitating disorders globally. They account for a substantial proportion of disability and mortality, with their incidence and burden continuing to rise in both developed and developing regions (Collaborators, 2019). These conditions not only impair motor, cognitive and sensory functions but also impose significant socioeconomic pressure through prolonged treatment needs, increased healthcare costs and reduced quality of life.

**FIGURE 1:**
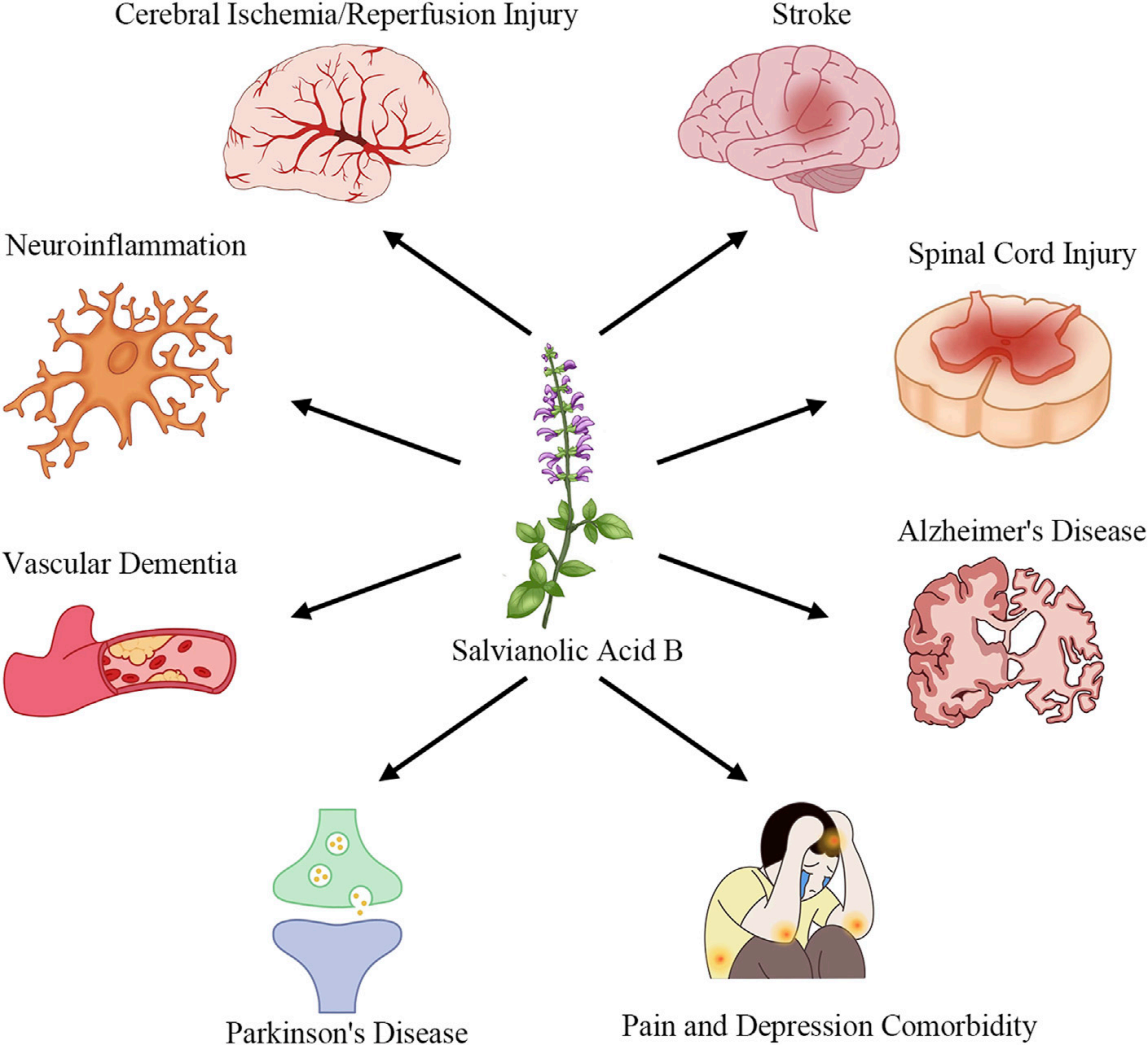
Applications of SalB in the treatment of neurological diseases.

Salvianolic Acid B (SalB), one of the primary water-soluble polyphenolic constituents of *Salvia miltiorrhiza* (Danshen), has attracted considerable interest due to its multifaceted pharmacological actions (Zhang et al., 2017). Preclinical studies have demonstrated that SalB exerts potent anti-inflammatory effects by inhibiting pro-inflammatory cytokine release and blocking TLR4/MyD88/NF-κB signaling (Wang et al., 2016; Zhang et al., 2016; Pei et al., 2023). It also scavenges reactive oxygen species and activates the Nrf2/ARE pathway to upregulate antioxidant enzymes such as HO-1 and NQO1 (Tang et al., 2014; Zhang et al., 2018; Xiao et al., 2020). In neuronal models, SalB preserves mitochondrial integrity, inhibits apoptosis via modulation of Bcl-2 and caspase-3, and promotes neurotrophic factor expression (Lee et al., 2013; Ling et al., 2017; He et al., 2018). Furthermore, SalB enhances angiogenesis and vascular repair by upregulating VEGF and STC1, thereby improving perfusion and supporting neurogenesis (Duan et al., 2019; Bi et al., 2022; Chen et al., 2022), and it inhibits platelet activation and thrombosis formation to protect the neurovascular unit (Xu et al., 2015; Guo et al., 2022; Neves et al., 2024). As introduced in this section, Figure 1 showcases how SalB’s therapeutic applications extend across multiple neurological disease domains.

In recent years, there has been a marked surge in investigations elucidating SalB’s mechanisms across diverse *in vitro* and *in vivo* neurological models, yet a comprehensive synthesis of these findings remains lacking. Moreover, challenges such as SalB’s limited bioavailability and chemical stability have spurred the development of novel delivery systems—from blood–brain barrier-permeable nanoparticles to injectable hydrogels—that warrant systematic appraisal. Against this backdrop, a focused review is both timely and necessary to integrate anti-inflammatory, antioxidant, anti-apoptotic, and pro-angiogenic pathways, examine emerging targets in neurodegeneration (e.g., proteinopathy modulation), and evaluate advanced formulation strategies designed to enhance SalB’s therapeutic potential.

To ensure a comprehensive overview, we systematically retrieved all relevant literature through PubMed using the search terms “Salvianolic Acid B” and specific neurological disease names within the title field, encompassing studies from all publication years. While preclinical and experimental studies have provided valuable insights into the therapeutic potential of SalB, clinical research data remain scarce. This review therefore focuses on summarizing the current preclinical and experimental evidence of SalB in the treatment of neurological diseases, with an emphasis on the mechanisms of action and potential therapeutic effects.

## 2 Application of SalB in the treatment of neurological diseases

### 2.1 Cerebral ischemia/reperfusion injury

Cerebral ischemia/reperfusion injury (CIRI) is the process in which the restoration of blood flow after an ischemic stroke paradoxically worsens brain tissue damage. This process involves complex pathophysiological processes such as oxidative stress, inflammation, and cell apoptosis (Pei et al., 2023). SalB reduces CIRI via several routes, providing novel therapy options for ischemic strokes.

Research has indicated that experimental animals treated with SalB have lower infarct sizes and improved neurological recovery after CIRI (Zhu et al., 2013). Besides, SalB prevents neuronal apoptosis, which protects neurons from ischemia reperfusion damage (Wang et al., 2016). These protective benefits are intimately related with SalB’s antioxidant and anti-inflammatory characteristics. Oxidative stress, a key factor in the pathophysiology of CIRI, causes lipid peroxidation, protein oxidation, and DNA damage. SalB reduces brain tissue damage by increasing the activity of antioxidant enzymes including superoxide dismutase (SOD) and glutathione peroxidase (GSH-Px), which reduces the production of oxidative stress products (Zhang et al., 2018). Moreover, SalB boosts its antioxidant efficiency by upregulating the expression of antioxidant genes, such as heme oxygenase-1 (HO-1) (Fan et al., 2018). Inflammation plays a critical role in the pathophysiology of CIRI, causing disruption of the blood-brain barrier (BBB), edema formation, and neuronal death. SalB reduces brain tissue damage by inhibiting the synthesis of inflammatory cytokines such as TNF-α, IL-1β, and IL-6, as well as reducing inflammatory cell infiltration (Wang et al., 2016). Besides, SalB has an additional anti-inflammatory impact via blocking the TLR4-mediated inflammatory signaling pathway (Zheng et al., 2023).

SalB’s preventive effects on CIRI are mediated by a variety of mechanisms. Research has discovered that SalB stimulates the production of vascular endothelial growth factor (VEGF), boosting angiogenesis and repair, and therefore improving blood flow to ischemic brain tissue (Duan et al., 2019). In addition, SalB decreases microcirculation barriers during ischemia/reperfusion by suppressing platelet activation and thrombosis (Guo et al., 2022).

Collectively, these findings underscore SalB’s multifaceted neuroprotective effects in cerebral ischemia/reperfusion injury, mediated by antioxidant defense enhancement, inflammatory cytokine suppression, and microvascular integrity preservation. Detailed mechanisms and experimental evidence are summarized in Table 1. While preclinical models consistently demonstrate efficacy, translational gaps persist due to the absence of human trials and unresolved questions regarding direct molecular interactions underlying these effects.

**TABLE 1:** Comparative pharmacological parameters and neuroprotective effects of SalB in CIRI models.

Study reference	Species	Administration route	Timing of administration	Key findings/mechanisms
Zhu et al. (2013)	Rats (Sprague-Dawley rats)	Intraperitoneal (i.p.)	9 h after reperfusion	SMND-309, a derivative of SalB, protected against cerebral ischemia/reperfusion injury by targeting the JAK2/STAT3 pathway, reducing infarct size, and improving neurological function
Wang et al. (2016)	Rats (Wistar rats)	Intraperitoneal (i.p.)	Immediately after reperfusion	SalB reduced infarct volume and improved neurological function via anti-inflammatory, anti-oxidative stress, and pro-angiogenic pathways. It prevented neuronal apoptosis and protected neurons from ischemia-reperfusion damage
Duan et al. (2019)	Rats (Wistar rats)	Intraperitoneal (i.p.)	1 h after reperfusion	The combination of SalB and borneol improved neurological function and reduced infarct size, possibly through modulation of metabolic pathways and enhancement of angiogenesis
Fan et al. (2018)	Rats (Wistar rats)	Intraperitoneal (i.p.)	5 consecutive days before reperfusion, once daily (prophylactic administration); treatment group administered once immediately after reperfusion	SalB boosted its antioxidant efficiency by upregulating the expression of antioxidant genes, such as heme oxygenase-1 (HO-1), thereby reducing oxidative stress and brain tissue damage
Zhang et al. (2018)	Mice (C57BL/6 mice)	Intraperitoneal (i.p.)	Immediately after reperfusion	SalB reduced brain tissue damage by increasing the activity of antioxidant enzymes including superoxide dismutase (SOD) and glutathione peroxidase (GSH-Px), which reduces the production of oxidative stress products
Zheng et al. (2023)	Mice (CD-1 mice)	Intraperitoneal (i.p.)	Immediately after reperfusion	SalB had an additional anti-inflammatory impact via blocking the TLR4-mediated inflammatory signaling pathway, reducing the synthesis of inflammatory cytokines such as TNF-α, IL-1β, and IL-6
Guo et al. (2022)	Rats (Sprague-Dawley rats) and Mice (C57BL/6 mice, including Nrf2 knockout mice)	Intraperitoneal (i.p.)	1 h after SAH induction, then once daily until sacrifice	SalB decreased microcirculation barriers during ischemia/reperfusion by suppressing platelet activation and thrombosis, thereby improving blood flow to ischemic brain tissue

### 2.2 Stroke

Stroke, an acute cerebrovascular condition, occurs when blood flow to the brain is suddenly interrupted, causing brain tissue damage (Hilkens et al., 2024). Ischemic stroke is often caused by vascular blockage, whereas hemorrhagic stroke results from brain vessel rupture.

SalB has been shown to minimize infarct size and enhance neurological function after stroke. In one research, the combination of SalB and ginsenoside Rg1, delivered intravenously, dramatically lowered the infarct size in rats with acute ischemic stroke and improved neurological behavior as judged by the Longa score and Left-Biased Swings test (Fu et al., 2021). Another study discovered that SalB decreased the infarct amount in a permanent middle cerebral artery occlusion (pMCAO) animal model, whose result was strongly connected to its antioxidant and anti-inflammatory capabilities (Lee et al., 2023).

SalB’s capacity to maintain the BBB further demonstrates its therapeutic effect on stroke. Disruption of the BBB is a significant cause of post-stroke edema and inflammatory reactions. SalB has been demonstrated to reduce the activity of matrix metalloproteinase-2 (MMP-2) and matrix metalloproteinase-9 (MMP-9), two important enzymes that impair BBB integrity (Fu et al., 2021). SalB also has neuroprotective effects via regulating numerous signaling pathways. For example, SalB can promote autophagy and death by blocking the AKT/mTOR signaling pathway, therefore decreasing the damage produced by stroke (Guo and Wang, 2022). Studies have demonstrated that the combination of SalB and ginsenoside Rg1 has a strong synergistic impact in stroke therapy. This synergy not only increases the neuroprotective impact but also decreases the dosage of individual drugs, minimizing the risk of adverse effects (Shen et al., 2024). Additionally, combination treatment with SalB and mesenchymal stem cells has been found to more effectively reduce CIRI and enhance neurological function recovery (Yan et al., 2023).

Notably, while preclinical evidence robustly supports SalB’s capacity to mitigate stroke-induced brain damage and neurological deficits, its therapeutic potential remains constrained by reliance on animal models and limited exploration of combination therapies’ mechanistic synergies. Critical unknowns include dose optimization and long-term safety profiles in human populations.

### 2.3 Spinal cord injury

Spinal cord injury (SCI) is a severe disorder of the central nervous system that is frequently accompanied by the breakdown of the blood–spinal cord barrier (BSCB), resulting in spinal cord edema and neuron damage (Li et al., 2024). Fan et al. observed that SalB dramatically lowered the water content of spinal cord tissue and the permeability of BSCB by producing SCI model in rats, therefore reducing spinal cord edema and enhancing the recovery of motor function (Fan et al., 2013). SalB plays an anti-inflammatory role by reducing the production of inflammatory markers TNF-α and NF-κ B. Tight junction protein is a key component of blood-brain barrier and blood-spinal barrier (BSCB). SalB can increase the expression of tight junction protein ZO-1 and occludin in spinal cord tissue of rats with SCI. In addition, treatment with the HO-1 inhibitor ZnPP partially reversed the protective effects of SalB. Specifically, ZnPP treatment significantly reduced the upregulation of ZO-1 and occludin (P < 0.05) and partially reversed the decrease in BSCB permeability, as measured by Evans Blue extravasation (P < 0.05). These findings indicate that the protective effects of SalB on BSCB integrity were significantly attenuated by ZnPP, suggesting the crucial role of the HO-1 pathway in mediating SalB’s effects.

Fu et al. discovered that SalB may considerably reduce spinal cord edema and infarct volume while also improving motor abilities in a rat SCI model (Fu et al., 2014). SalB’s oxidative stress capacity allows it to efficiently prevent oxidation product production while also maintaining antioxidant enzyme activity. Furthermore, SalB can lengthen the activation time of extracellular signal-regulated kinase (ERK), and the neuroprotective effect of SalB is partially diminished when the ERK inhibitor PD98059 is used, indicating that SalB may exercise its neuroprotective impact via activating the ERK pathway.

Cumulatively, these data highlight SalB’s potential to mitigate secondary injury cascades in spinal cord trauma, particularly through barrier stabilization and anti-inflammatory modulation. However, the transition to clinical application is hindered by the exclusive reliance on rodent models and absence of biomarkers predicting therapeutic response or adverse effects in humans.

### 2.4 Alzheimer’s disease

Alzheimer’s Disease (AD) is a neurodegenerative condition that includes β-amyloid accumulation, neurofibrillary tangles, neuronal death, and cognitive impairment. As the world’s population ages, the prevalence of Alzheimer’s disease is increasing, providing a significant public health concern (Scheltens et al., 2021).

SalB has been shown to have neuroprotective effects in several AD models. In the PC12 neuronal cell model, SalB substantially decreased Aβ42 fibrillation and counteracted its harmful effects on neuronal cells (Tang and Zhang, 2001). In the *Drosophila melanogaster* AD model, SalB dramatically reduced Aβ toxicity and improved cognitive performance in the flies (Tan et al., 2021). SalB inhibits the inflammatory response and oxidative stress caused by Aβ25-35 peptide in AD animal models, reducing cognitive impairment and neuronal damage (Lee et al., 2013).

The neuroprotective mechanisms of SalB in AD involve multiple aspects. Firstly, it can inhibit the activity of BACE1, a key enzyme in Aβ production. In the SH-SY5Y-APPsw cell model, SalB significantly reduced the levels of Aβ40 and Aβ42, which was closely related to its inhibitory effect on BACE1 (Tang et al., 2016). Secondly, SalB possesses anti-inflammatory and antioxidant properties, capable of inhibiting microglial activation, reducing the release of inflammatory cytokines, and lowering oxidative stress levels, thereby mitigating neuroinflammation and oxidative damage in the pathological process of AD (Wang et al., 2023). Additionally, SalB can inhibit the fibrillation process of Aβ *in vitro*, further reducing its neurotoxicity (Tang and Zhang, 2001). Lastly, studies have also found that SalB may improve cognitive dysfunction in AD by modulating the cholinergic system, possibly through indirect effects on the γ-aminobutyric acid (GABA)ergic neurotransmitter system (Kim et al., 2011).

While SalB demonstrates encouraging neuroprotective activity across diverse AD models, its clinical relevance remains speculative. Key limitations include reliance on non-human systems, lack of comparative efficacy data against FDA-approved AD drugs, and incomplete elucidation of Aβ-independent mechanisms (e.g., cholinergic modulation specificity).

### 2.5 Pain and depression comorbidity

Pain and depression comorbidity is a common clinical phenomenon, with a high degree of co-occurrence between the two conditions, severely impacting patients’ quality of life. Studies have shown that patients with chronic pain are at a significantly increased risk of developing depression, and individuals with depression are also more prone to experiencing pain symptoms (Llorca-Torralba et al., 2022). This comorbid condition complicates therapy and may lead to poor therapeutic results and prognosis.

In mouse forced-swim and tail-suspension tests, Feng et al. reported a significant reduction in immobility time after treatment with SalB, with effects comparable to the classic antidepressant imipramine (Feng et al., 2012). Specifically, SalB at doses of 5, 10, and 20 mg/kg demonstrated a marked decrease in immobility, suggesting a robust antidepressant-like effect. Additional study has demonstrated that SalB can restore depressive-like behaviors in a chronic moderate stress (CMS) mouse model, such as decreased sucrose preference and increased immobility duration in the forced swim and tail suspension tests (Zhang et al., 2016). SalB modulates neuroinflammatory pathways by inhibiting pro-inflammatory cytokines IL-1β and TNF-α and elevating anti-inflammatory cytokines IL-10 and TGF-β, leading to improved depression symptoms. Also, SalB has been found to have therapeutic effects on pain-depression comorbidity. Liu et al.'s study demonstrated that SalB can alleviate chronic restraint stress (CRS)-induced depressive-like behavior comorbid with pain by inhibiting the excitation of GABAergic neurons in the amygdala (Liu et al., 2022). This mechanism involves the activation of the ERK-CREB-BDNF signaling pathway, through which SalB inhibits the overexcitation of GABAergic neurons, thereby relieving pain and depressive symptoms. This study not only reveals a new mechanism of SalB in the treatment of depressive-like behavior comorbid with pain but also provides a new target for the development of novel antidepressant and analgesic drugs.

Furthermore, in a separate chronic mild stress (CMS) model, Huang et al. revealed that SalB curtailed NLRP3 inflammasome activation, accompanied by reductions in IL-1β and ROS levels (Huang et al., 2018). The treatment also led to a notable attenuation of depressive-like behaviors, including improved sucrose consumption and reduced immobility in behavioral tests.

These insights position SalB as a multifunctional candidate for managing comorbid pain and depression, leveraging neuroinflammatory and GABAergic pathway modulation. Yet, mechanistic overlap with existing antidepressants/analgesics and the absence of human trials preclude definitive conclusions about its therapeutic superiority or safety profile.

### 2.6 Parkinson’s disease

Parkinson’s disease (PD) is a chronic neurodegenerative disorder caused by the degeneration of dopaminergic neurons in the substantia nigra (Bloem et al., 2021). Zhou et al. discovered that SalB exhibits neuroprotective effects in Parkinson’s disease (PD) models by modulating glial cells via the Nrf2 pathway (Zhou et al., 2014). *In vitro* studies demonstrated that SalB, at concentrations of 50 μM and 100 μM, protected dopaminergic neurons from MPP + - and LPS-induced toxicity, as evidenced by a reduction in LDH release and an increase in [^3^H]DA uptake. Furthermore, SalB dose-dependently inhibited microglial pro-inflammatory cytokine release, including TNF-α and IL-1β, and decreased NO production. Additionally, SalB enhanced astrocytic GDNF expression, both at the mRNA and protein levels. Western blot analysis revealed SalB-induced Nrf2 expression and nuclear translocation, and Nrf2 knockdown partially reversed these protective effects. In MPTP-treated mice, SalB pretreatment attenuated dopaminergic neuronal loss, inhibited neuroinflammation, increased GDNF expression, and improved neurological function, consistent with its *in vitro* effects.

While SalB’s neurorescue effects in PD models are compelling, particularly through Nrf2-dependent glial regulation, clinical advancement is impeded by reliance on toxin-based animal paradigms and unaddressed questions about long-term dopaminergic neuron preservation in human neurodegeneration.

### 2.7 Vascular dementia

Vascular dementia (VD) is the second most common type of dementia after AD, accounting for approximately 15% of dementia cases (O'Brien and Thomas, 2015). It is primarily caused by cerebrovascular diseases that lead to impaired brain function and subsequent cognitive impairment. Recent studies have shown that SalB, through its multiple pharmacological effects, significantly improves cognitive impairment in rat models of VD (Ma et al., 2017). Specifically, SalB treatment upregulated hippocampal IGF-1 expression, as evidenced by Western blotting and ELISA, which was significantly lower in VD model rats compared to controls (p < 0.05). IGF-1, as a key factor in nervous system development and protection, its elevated expression may promote neuron survival and synaptic plasticity. Furthermore, in the Morris water maze test, SalB-treated VD rats exhibited a shortened escape latency and increased number of platform crossings in the probe trial, compared to VD controls (p < 0.05 for both), indicating improved spatial learning and memory. Additionally, SalB promotes neuron survival and reduces apoptosis by activating the Akt signaling pathway, particularly by increasing the level of phosphorylated Akt (p-Akt), thereby further improving cognitive function in VD rats.

In summary, SalB’s cognitive-enhancing effects in vascular dementia models—driven by neurotrophic (IGF-1) and pro-survival (Akt) signaling—warrant further investigation. However, the exclusive use of surgical vascular occlusion models and lack of co-morbidity analysis (e.g., hypertension, diabetes) limit extrapolation to heterogeneous human VD populations.

### 2.8 Neuroinflammation

Neuroinflammation, as a core pathological process in various neurological diseases, involves the activation of immune cells, the release of inflammatory factors, and neuronal damage, posing a serious threat to the normal function of the nervous system (Yan et al., 2018).

The inhibitory effect of SalB on neuroinflammation is firstly manifested in its regulation of immune cell activity. In the nervous system, microglia and astrocytes, as the main immune cells, play a crucial role in the process of neuroinflammation. SalB can inhibit the overactivation of these cells, reducing the release of inflammatory factors such as IL-1β and TNF-α, thereby effectively alleviating the degree of neuroinflammation (Liu et al., 2020; Zhao et al., 2023a). SalB also exerts anti-inflammatory effects by regulating specific signaling pathways. The NLRP3 inflammasome is an important target in neuroinflammation, and its activation leads to the massive release of inflammatory factors and neuronal damage. SalB can inhibit the activation of the NLRP3 inflammasome, blocking this inflammatory pathway and thus protecting neurons from damage (Jiang et al., 2017). In addition, SalB enhances the antioxidant and anti-inflammatory capabilities of neurons by promoting the expression of the SIRT1 signaling pathway, providing additional support for neuroprotection (Xia et al., 2023).

SalB also indirectly exerts neuroprotective effects through other mechanisms. For example, in neuroinflammation induced by endoplasmic reticulum stress, SalB can inhibit the activation of the NLRP3 inflammasome and pyroptosis through the AMPK/FoxO4 and Syndecan-4/Rac1 signaling pathways, thereby reducing neuronal damage (Tang et al., 2022). This finding reveals the multi-target mechanism of SalB in coping with complex neuroinflammatory environments, further enhancing its potential as a neuroprotective agent. Furthermore, SalB has also demonstrated significant anti-inflammatory effects in CIRI. By inhibiting platelet activation and the CD40/NF-κB signaling pathway, SalB can reduce the production of neuroinflammatory mediators after cerebral ischemia, thereby alleviating brain damage and promoting the recovery of neurological function (Xu et al., 2017).

In aggregate, these studies underscore SalB’s multifaceted anti-inflammatory actions in neuroinflammation, encompassing immune cell modulation, inflammatory pathway blockade, and cytoprotective signaling. Despite compelling preclinical evidence across models of neuroinflammation, therapeutic development is constrained by the absence of human trials and incomplete mechanistic dissection of pathway-specific contributions to neuroprotection.

## 3 Mechanistic study of SalB in the treatment of neurological diseases

### 3.1 Anti-inflammatory and anti-oxidative stress effects

SalB has garnered widespread attention in recent years in the field of anti-inflammatory treatment for neurological diseases. Its anti-inflammatory mechanisms exhibit multidimensional and multi-target characteristics.

In studies on CIRI, SalB pretreatment significantly reduced the expression levels of inflammatory factors such as TNF-α, IL-1β, and ICAM-1 in ischemic brain tissue. This effect is closely related to SalB’s ability to inhibit platelet activation and reduce the release of inflammatory mediators (Xu et al., 2015; Jiang et al., 2017). Additionally, it has been observed that SalB regulates the function of microglia, effectively inhibiting their excessive activation, thereby alleviating neuroinflammatory responses (Wang et al., 2010). Further research has revealed that SalB also exerts anti-inflammatory effects by modulating multiple signaling pathways. Specifically, SalB can inhibit the activation of the NF-κB signaling pathway, which is crucial for the transcription of various inflammatory factors (Huang et al., 2018; Zhao et al., 2023b). Furthermore, SalB regulates endothelial cell function through signaling pathways such as AMPK/FoxO4 and Syndecan-4/Rac1, thereby inhibiting the production of inflammatory mediators (Tang et al., 2022).

Experiments have shown that SalB can inhibit Aβ-induced microglia activation, reduce the activation of the NLRP3 inflammasome, and decrease the release of downstream inflammatory factors IL-1β and IL-18 (Zhao et al., 2023a). In a subarachnoid hemorrhage (SAH) model, SalB also improved neuroinflammation and neuronal damage by blocking the NLRP3 inflammasome (Xia et al., 2023). In a CMS model of depression, SalB treatment significantly reduced the expression of multiple inflammatory factors in the hippocampus, thereby improving depressive-like behavior (Huang et al., 2018). In a model of neuropathic pain induced by SCI, SalB reduced the release of inflammatory mediators by inhibiting the TLR4/MyD88 signaling pathway, effectively alleviating pain (Wang et al., 2019).

In parallel with its anti-inflammatory effects, SalB demonstrates significant antioxidant activity. SalB’s phenolic hydroxyl group structure provides hydrogen atoms that stabilize free radicals, preventing lipid peroxidation and lowering the formation of oxidative stress products (Xiao et al., 2020). In neuronal injury models, SalB considerably decreases the levels of oxidative stress indicators such as malondialdehyde (MDA) and increases the activity of SOD, demonstrating its good antioxidant impact (Jiang et al., 2017). Furthermore, SalB enhances cellular antioxidant capacity by upregulating the expression of antioxidant enzymes such as HO-1 (Xiao et al., 2020).

In PD models, SalB has been shown to significantly alleviate neuronal damage induced by 1-methyl-4-phenylpyridinium ion (MPP+). This protective effect is partly due to SalB’s anti-oxidative stress ability, which reduces the generation of reactive oxygen species (ROS), inhibits the drop in mitochondrial membrane potential, and so preserves mitochondrial function (Zhao et al., 2021). SalB can also further enhance cellular antioxidant defense by activating the AMPK signaling pathway and upregulating the expression of Sirtuin3 (Zhao et al., 2021).

SalB has been shown in CIRI tests to have substantial anti-oxidative stress properties. It decreases the ROS burst during ischemia-reperfusion, suppresses oxidative stress-induced cell death, and thereby lowers brain tissue damage (Tang et al., 2014). SalB can also promote the expression of antioxidant proteins such as NQO1 and HO-1 by activating the Nrf2 signaling pathway, further enhancing cellular antioxidant capacity (Lv et al., 2015). In addition, SalB exhibits anti-oxidative stress effects in depression models. Studies have found that SalB can decrease the levels of MDA and 4-hydroxynonenal (4-HNE) in the hippocampus of rats with depression-like behavior, while increasing the activity of catalase (CAT), thereby reducing oxidative stress-induced damage to neurons (Liao et al., 2020). These antioxidant effects may be related to SalB’s regulation of the AMPK/SIRT1 signaling pathway, which plays an important role in anti-oxidative stress (Liao et al., 2020).

### 3.2 Neuroprotective effects

The neuroprotective mechanisms of SalB are intricate and multifaceted, involving several aspects such as reduction of neuronal apoptosis, promotion of neural regeneration, and improvement of mitochondrial function. Firstly, SalB has been proven to significantly alleviate neuronal damage in various neurological disorders. In the CIRI model, SalB mitigates brain tissue edema and improves neurological function scores by inhibiting cellular apoptosis (Ling et al., 2017; Wang and Hu, 2018). Furthermore, SalB demonstrates neuroprotective effects in AD treatment by suppressing Aβ-induced neuronal apoptosis and inflammatory responses, thereby effectively slowing down cognitive decline in AD mouse models (Lee et al., 2013).

One of the key neuroprotective mechanisms of SalB is its ability to protect against mitochondrial dysfunction, particularly in the context of Aβ-induced toxicity in an *in vitro* Alzheimer’s disease model. Studies have systematically applied mitochondrial assays, including measurements of mitochondrial superoxide production, membrane potential, and ATP production, to demonstrate that SalB can ameliorate Aβ-induced mitochondrial dysfunction. Specifically, SalB reduces mitochondrial swelling, inhibits mitochondrial membrane potential depletion, and restores the activity of mitochondrial respiratory chain complexes, thus protecting neurons from oxidative stress damage (He et al., 2018). While the current data primarily supports these findings in the Aβ-induced toxicity model, it is noteworthy that in the CIRI model, SalB also effectively alleviates neuronal oxidative stress damage by enhancing mitochondrial antioxidant enzyme activity and inhibiting mitochondrial membrane potential collapse, as indicated by changes in mitochondrial function-related parameters (Ling et al., 2017). However, further systematic studies are warranted to confirm the protective effects of SalB on mitochondrial function across different neurological disease models.

Additionally, SalB exerts neuroprotective effects by modulating multiple signaling pathways. Research has found that SalB activates the IGF-1/Akt signaling pathway, leading to increased expressions of IGF-1 and p-Akt in the hippocampus of VD model rats, which in turn inhibits neuronal apoptosis and ameliorates cognitive dysfunction (Ma et al., 2017). In PD models, SalB, through an Nrf2-dependent pathway, alleviates neuroinflammation mediated by glial cell activation and induces astrocytic activation, increasing the expression of GDNF, thereby protecting dopaminergic neurons (Zhou et al., 2014).

Moreover, SalB has been found to promote neural regeneration. By upregulating the expression of stanniocalcin 1 (STC1), SalB facilitates angiogenesis and neural regeneration in cerebral ischemic rats, thereby mitigating neurological injury (Bi et al., 2022). STC1, as a multifunctional secretory protein, plays a crucial role in angiogenesis and neuroprotection. Findings from that study suggest that SalB may enhance STC1 expression through activation of the mTOR and AKT signaling pathways, as indicated by increased phosphorylation of these kinases in ischemic brain tissue and HUVECs. However, this proposed mechanism still requires validation in other experimental models.

### 3.3 Angiogenesis and vascular repair

In the treatment of neurological diseases, angiogenesis and repair represent a crucial process that not only provides essential nutrients and oxygen to damaged neural tissues but also participates in neural regeneration and functional recovery. SalB has demonstrated significant effects in promoting angiogenesis and repair through various mechanisms.

SalB, in synergy with ferulic acid (FA), markedly enhances angiogenesis in human umbilical vein endothelial cells (HUVECs) and zebrafish by modulating VEGF signaling (Chen et al., 2022). This synergistic action not only amplifies the expression of VEGF and its receptors but also fosters endothelial cell proliferation, migration, and tube formation, laying the foundation for neovascularization. Furthermore, SalB mitigates high glucose-induced disruption of brain microvascular endothelial cells, thereby preserving the integrity of the BBB and promoting angiogenesis through the ROS/HIF-1α/VEGF and miR-200b/VEGF signaling pathways (Yang et al., 2016). In hyperglycemic conditions, SalB inhibits ROS production, reduces HIF-1α degradation, and upregulates VEGF expression, ultimately facilitating neovascularization. Concurrently, SalB enhances vascular generation by upregulating miR-200b expression, which indirectly inhibits VEGF degradation.

Beyond direct angiogenesis, SalB also promotes tissue repair and regeneration by modulating immune cell migration and caveolin-1 (Cav1)-mediated blastema formation (Qin et al., 2024). In a zebrafish tail fin regeneration model, SalB facilitates dynamic immune cell aggregation and regression, minimizing inflammatory cell infiltration and fostering a conducive microenvironment for angiogenesis. Besides, SalB upregulates Cav1 expression, promoting blastema formation and neovascularization, thus accelerating tissue repair. Moreover, an injectable hydrogel composed of hyaluronic acid (HA) and gelatin, combined with SalB and VEGF, introduces novel material support for angiogenesis and repair in neurological diseases (Zhou et al., 2024). This hydrogel exhibits excellent biocompatibility and biodegradability, enabling sustained release of SalB and VEGF, which stimulates stem cell proliferation and differentiation, as well as neovascularization. In a mouse model of traumatic brain injury, injection of the hydrogel containing SalB and VEGF significantly reduced lesion volume and promoted brain tissue repair and neovascularization.

### 3.4 Inhibition of platelet activation and thrombus formation

In the treatment of neurological diseases, SalB not only exhibits extensive biological activities but also demonstrates a remarkable inhibitory effect on platelet activation and thrombosis, which is of significant importance for the prevention and treatment of neurological diseases closely related to thrombosis.

SalB has been proven to effectively suppress platelet activation and aggregation. Studies have shown that SalB can dose-dependently inhibit platelet aggregation induced by ADP or α-thrombin, and significantly reduce the release of soluble P-selectin, indicating its potent inhibitory effect on platelet activation *in vitro* (Xu et al., 2015). Furthermore, SalB directly blocks the catalytic site of thrombin, thereby inhibiting its activity, which constitutes a crucial aspect of its anti-thrombotic mechanism (Neves et al., 2024). Further research has revealed the molecular mechanisms underlying SalB’s inhibition of platelet activation and thrombosis. SalB has been shown to inhibit the activation of platelet surface P2Y1, P2Y12 receptors, and α2β1 integrins, which are crucial for platelet activation (Xu et al., 2015). Additionally, SalB can inhibit the activation of NF-κB, thereby reducing the endothelial cell inflammation induced by activated platelets, and indirectly suppressing further platelet activation and thrombosis (Ba et al., 2014). NF-κB is a key inflammatory transcription factor whose activation promotes the expression of various inflammatory cytokines, thus increasing the risk of thrombosis.

SalB’s antithrombotic activity is not confined to its direct influence on platelets; it also protects vascular endothelial cells. SalB has been reported to diminish the increase in endothelial cell permeability generated by VEGF, which is achieved by upregulating the expression of tight junction proteins (such as occludin and claudin-5) and downregulating the expression of caveolins (such as caveolin-1 and caveolin-2) (Ba et al., 2014).

### 3.5 Regulation of signaling pathways

SalB, as a compound with extensive therapeutic potential and diverse biological activities, has demonstrated significant efficacy in the treatment of neurological diseases. Its pivotal role lies in the ability to modulate multiple signaling pathways, enabling precise regulation of key processes such as neuroprotection, anti-inflammation, and antioxidant stress.

Firstly, in terms of neuroprotection, SalB exerts significant effects by activating distinct signaling pathways. In the treatment of depression and comorbid pain, SalB activates the ERK-CREB-BDNF signaling pathway, promoting the expression of BDNF, which subsequently inhibits the overexcitation of GABAergic neurons, thereby alleviating depressive-like behaviors and pain comorbidity (Liu et al., 2022). In cerebral small vessel disease (CSVD), SalB upregulates the expression of STAT3 and VEGF, activating the STAT3/VEGF signaling pathway, which promotes angiogenesis and neural repair, leading to improved cognitive function (Wang and Hu, 2018). Furthermore, SalB activates the SIRT1 signaling pathway, inhibiting cell apoptosis and inflammation, thus demonstrating remarkable neuroprotective effects in stroke treatment (Lv et al., 2015). In experimental SAH, SalB reduces brain edema, neuronal death, and neurological dysfunction through the activation of Nrf2-and SIRT1-dependent pathways (Zhang et al., 2018).

Secondly, SalB exhibits outstanding performance in neural regeneration and repair. Through the PI3K/Akt signaling pathway, SalB directly stimulates the proliferation of adult neural stem/progenitor cells (NSPCs), providing robust support for neural regeneration (Zhuang et al., 2012). In the neural differentiation of induced pluripotent stem cells (iPSCs), SalB significantly promotes the differentiation of iPSCs into neural stem cells and further into neurons via the PI3K/AKT/GSK3β/β-catenin pathway (Shu et al., 2018).

In terms of anti-inflammation and antioxidant stress, SalB also displays notable effects. It significantly promotes the polarization of microglial cells towards the M2 phenotype in stressed mice while inhibiting the expression of M1 polarization-related inflammatory factors, a mechanism involving the regulation of microglial cell polarization status (Zhang et al., 2017). In addition, SalB inhibits the activation of the JAK/STAT1 pathway in endothelial cells induced by IFN-γ, exerting a potent anti-inflammatory effect (Chen et al., 2011). In antioxidant stress responses, SalB activates the IGF-1/Akt pathway, inhibiting neuronal apoptosis and reducing oxidative stress, thereby improving cognitive dysfunction in VD rats (Ma et al., 2017). It also resists oxidative stress-mediated dysfunction in human endothelial progenitor cells (EPCs), supporting angiogenesis and repair (Tang et al., 2014).

Moreover, SalB plays a unique role in the treatment of specific neurological diseases such as PD. By inhibiting oxidative stress and restoring mitochondrial function, SalB provides protection against MPP + -induced neuronal damage (Zhao et al., 2021).

## 4 Synergistic drugs of SalB and effects

SalB not only has individual pharmacological effects in the treatment of neurological illnesses, but it also has strong synergistic effects with other medications or components, which increases its therapeutic efficacy. The mechanisms causing these synergistic effects are complicated and diverse, including various signaling pathways and biological processes, and offer fresh perspectives on neurological illness treatment.

In the treatment of CIRI, the synergistic effect of SalB and ginsenoside Rg1 considerably improves the therapeutic potential for ischemic stroke. SalB and Rg1 combination therapy has been found in studies to reduce infarct volume while also improving neurobehavioral function and increasing the number of neurons, with benefits superior to SalB or Rg1 alone (Shen et al., 2024). Further mechanistic studies suggest that this synergistic effect may be related to the regulation of glycerophospholipid metabolism pathways, indicating a metabolic-level synergy between SalB and Rg1 (Shen et al., 2024). Additionally, the combination of SalB and puerarin also exhibits significant synergistic effects in CIRI. This combination not only reduces oxidative stress and cell apoptosis but also improves mitochondrial membrane potential, thereby alleviating CIRI (Ling et al., 2018). Mechanistically, this synergistic effect may be associated with the inhibition of the TLR4/MyD88 signaling pathway and the activation of the SIRT1 signaling pathway, which play crucial roles in neuroinflammation and cell apoptosis (Ling et al., 2018). In terms of antioxidant and anti-inflammatory mechanisms, the synergistic effect of SalB and ginsenoside Re provides significant protection against Ox-LDL-induced endothelial cell apoptosis. Studies have demonstrated that the combination of SalB and Re can more effectively reduce the levels of inflammatory mediators, increase the activity of antioxidant enzymes, and restore the balance of cellular redox status by regulating the expression of receptors such as LOX-1, NOX4, and ERα, thereby inhibiting endothelial cell apoptosis (Yang et al., 2018).

SalB also exhibits synergistic potential with novel drugs or targets. As a novel type I IRE1 kinase inhibitor, SalB interacts with the active conformation of IRE1, not only inhibiting AngII-induced angiogenesis but also protecting vascular endothelial cells from hypoxia-induced damage (Fan et al., 2022). Thus, the synergistic mechanisms of SalB in the treatment of neurological diseases are diverse, including metabolic regulation, signaling pathway inhibition, antioxidant stress, and anti-inflammation. These synergistic effects not only enhance the therapeutic efficacy of SalB but also provide a theoretical basis for its combined use with other drugs or components.

## 5 Challenges and future perspectives

**FIGURE 2:**
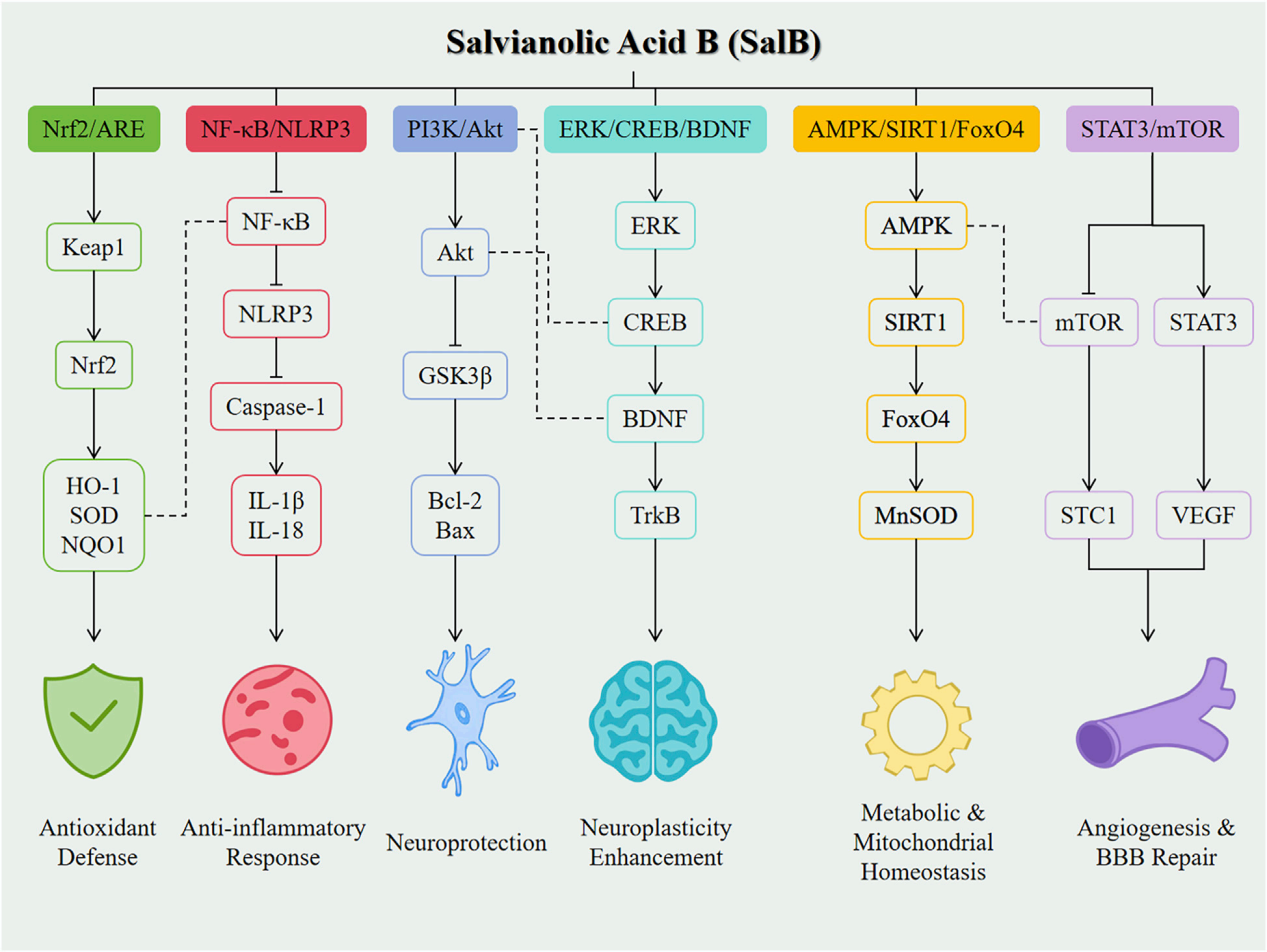
Mechanistic overview of SalB in neurological diseases. Line styles indicate the type of interaction: Solid arrows (→) represent direct activation between molecules. Bar-headed lines (⊣) represent direct inhibition. Plain dashed lines (---) indicate cross-talk or indirect regulatory interactions between different signaling pathways.

Despite the promising application prospects of SalB in the treatment of neurological diseases, its clinical translation still faces numerous challenges. Firstly, the issues of chemical stability and bioavailability of SalB need to be addressed urgently. Studies have shown that SalB is readily metabolized in the body and has low oral bioavailability, which limits its effective concentration and duration of action *in vivo* (Chen et al., 2006). To improve the stability and bioavailability of SalB, researchers are working on developing novel drug delivery systems, such as nanocarriers, to enhance the solubility, stability, and targeting of SalB (Grossi et al., 2016). For example, BBB-permeable nanoparticles, as potential carriers for SalB, have demonstrated good brain delivery efficiency and biocompatibility, providing new ideas for the application of SalB in the treatment of central nervous system diseases (Grossi et al., 2016). Secondly, the specific mechanisms underlying the therapeutic effects of SalB in neurological diseases have not been fully elucidated. As illustrated in Figure 2, SalB exerts its neuroprotective effects through coordinated regulation of multiple signaling pathways in nervous system disorders. Although studies have revealed multiple mechanisms of action for SalB, including anti-inflammatory, antioxidant stress, neuroprotective, angiogenic and repair, inhibition of platelet activation and thrombosis, and regulation of signaling pathways (Kong et al., 2022; Bi et al., 2023), the interactions between these mechanisms and their specificity in different disease states require further exploration. In particular, how SalB exerts its neuroprotective effects by modulating complex signaling pathway networks is an important direction for future research.

Furthermore, the synergistic effects of SalB in the treatment of neurological diseases also deserve attention. Studies have shown that the combination of SalB with other drugs or active ingredients can produce synergistic effects, thereby enhancing therapeutic efficacy (Kong et al., 2022). However, optimizing drug combinations, determining the optimal dose ratios, and exploring the molecular mechanisms of synergistic effects remain challenges to be addressed in future research. Future research directions also include the early intervention and long-term treatment effect evaluation of SalB in neurological diseases. Early intervention is of great significance for preventing or delaying disease progression, while the evaluation of long-term treatment effects helps to determine the safety and effectiveness of SalB. Meanwhile, individual differences and genetic backgrounds in the clinical application of SalB also need to be further considered to achieve precision medicine.

The current research on the effects of SalB on neurological diseases is summarized in Table 2. From the perspective of clinical application prospects, with a deeper understanding of the mechanisms of action of SalB and the development of novel drug delivery systems, SalB has the potential to become a new option for the treatment of neurological diseases. However, to achieve this goal, current challenges need to be overcome, and close integration of basic research and clinical translation should be strengthened to promote the widespread application of SalB in the field of neurological disease treatment. In this review, we have summarized the preclinical evidence supporting the therapeutic potential of SalB in various neurological diseases. However, it is important to acknowledge the significant methodological differences across the studies included in our analysis. These differences, including variations in experimental conditions, sample sizes, and model systems used, may affect the reliability and generalizability of the conclusions drawn from individual studies. Looking forward, it is crucial for future research to address these limitations. Standardized experimental protocols should be developed to ensure consistency across studies, allowing for more reliable comparisons and meta-analyses. Additionally, larger, well-designed preclinical studies are needed to confirm the therapeutic effects of SalB and to explore its potential synergistic effects with other drugs. Ultimately, the conduct of randomized controlled trials (RCTs) will be essential to evaluate the clinical efficacy and safety of SalB in various neurological conditions, paving the way for its potential translation into clinical practice. Future research on SalB should prioritize the design and execution of randomized controlled trials (RCTs) to assess its clinical efficacy and safety in various neurological conditions. Additionally, efforts should be made to address the challenges associated with the chemical stability and bioavailability of SalB, as well as to optimize drug delivery systems for enhanced therapeutic outcomes.

**TABLE 2:** Applications and mechanistic studies of SalB in various neurological diseases.

Neurological disease	Application of SalB	Mechanistic study of SalB	References
Cerebral ischemia/reperfusion injury (CIRI)	Reduces infarct volume, enhances neurological function, maintains blood-brain barrier integrity, regulates signaling pathways, inhibits neuronal apoptosis, reduces oxidative stress and inflammation	Inhibits inflammatory factors (TNF-α, IL-1β, ICAM-1), regulates microglia activity, inhibits NF-κB signaling pathway, scavenges free radicals, upregulates antioxidant enzymes (SOD, HO-1), stimulates VEGF production, improves mitochondrial function, inhibits platelet activation and thrombosis	Wang et al. (2016), Zhang et al. (2018), Fan et al. (2018), Xu et al. (2015), Ling et al. (2017), Wang and Hu (2018)
Stroke	Minimizes infarct size, enhances neurological function, maintains blood-brain barrier integrity, regulates signaling pathways	Inhibits inflammatory factors, regulates microglia activity, inhibits NF-κB signaling pathway, scavenges free radicals, upregulates antioxidant enzymes, stimulates VEGF production, improves mitochondrial function, inhibits platelet activation and thrombosis, activates autophagy via AKT/mTOR signaling pathway	Fu et al. (2021), Lee et al. (2023), Fu et al. (2021), Guo and Wang (2022), Shen et al. (2024), Yan et al. (2023)
Spinal cord injury (SCI)	Reduces edema, enhances motor function recovery, protects the blood-spinal cord barrier, regulates signaling pathways	Inhibits inflammatory factors (TNF-α, NF-κB), increases tight junction protein expression (ZO-1, occludin), activates ERK pathway, scavenges free radicals, upregulates antioxidant enzymes, improves mitochondrial function	Fan et al. (2013), Fu et al. (2014), Liu et al. (2020), Zhao et al. (2023a), Tang et al. (2022)
Alzheimer’s disease (AD)	Suppresses amyloid-beta formation and fibrillation, decreases neuroinflammation, regulates the cholinergic system, improves cognitive performance	Inhibits BACE1 activity, reduces Aβ levels, inhibits neuroinflammation and oxidative stress, modulates cholinergic system, activates SIRT1 signaling pathway	Tang et al. (2016), Wang et al. (2023), Kim et al. (2011), Tang and Zhang (2001)
Pain and depression comorbidity	Exhibits antidepressant and analgesic effects	Modulates neuroinflammatory pathways, inhibits pro-inflammatory cytokines (IL-1β, TNF-α), elevates anti-inflammatory cytokines (IL-10, TGF-β), inhibits GABAergic neuron excitation via ERK-CREB-BDNF signaling pathway, inhibits NLRP3 inflammasome activation	Zhang et al. (2016), Liu et al. (2022), Huang et al. (2018)
Parkinson’s disease (PD)	Protects dopaminergic neurons, acts as an antioxidant and anti-inflammatory	Modulates glial cells via Nrf2 pathway, inhibits MPP + - and LPS-induced toxicity, inhibits microglial pro-inflammatory cytokine release, enhances astrocytic GDNF expression, activates AMPK signaling pathway, upregulates Sirtuin3 expression	Zhou et al. (2014), Zhao et al. (2021)
Vascular dementia (VD)	Enhances cognitive performance, stimulates angiogenesis, inhibits apoptosis	Activates IGF-1/Akt signaling pathway, increases IGF-1 and p-Akt expression, inhibits neuronal apoptosis, promotes neuron survival, stimulates angiogenesis via VEGF signaling pathway	Ma et al. (2017), Wang and Hu (2018)
Neuroinflammation	Inhibits neuroinflammation, protects neurons from damage	Regulates immune cell activity (microglia, astrocytes), inhibits NLRP3 inflammasome activation, promotes SIRT1 signaling pathway, inhibits oxidative stress and inflammation, regulates AMPK/FoxO4 and Syndecan-4/Rac1 signaling pathways	Liu et al. (2020), Zhao et al. (2023a), Xia et al. (2023), Tang et al. (2022)

## 6 Conclusion

SalB has demonstrated significant therapeutic potential in preclinical models of neurological disorders, with its efficacy being most pronounced during acute/early disease phases (e.g., post-stroke reperfusion, pre-plaque Alzheimer’s disease, and acute spinal cord injury). However, challenges remain, such as poor bioavailability, rapid metabolism, and chemical instability, which limit its clinical application. While its mechanisms are well understood, direct comparisons with other neuroprotective therapies are lacking, and the interactions of SalB’s signaling pathways need further exploration. Clinical trials are essential to assess its safety, optimal dosing, and long-term efficacy. Key steps for clinical translation include developing advanced drug delivery systems to improve bioavailability, conducting large-scale preclinical studies, and initiating well-controlled randomized clinical trials (RCTs) to bridge the gap between laboratory findings and clinical use.
